# Heat-stress-modulated induction of NF-κB leads to brucellacidal pro-inflammatory defense against *Brucella abortus* infection in murine macrophages and in a mouse model

**DOI:** 10.1186/s12866-018-1185-9

**Published:** 2018-05-25

**Authors:** Huynh Tan Hop, Lauren Togonon Arayan, Alisha Wehdnesday Bernardo Reyes, Tran Xuan Ngoc Huy, Won Gi Min, Hu Jang Lee, Man Hee Rhee, Hong Hee Chang, Suk Kim

**Affiliations:** 1 Institute of Animal Medicine, College of Veterinary Medicine, Gyeongsang National University, Jinju, 52828 Republic of Korea; 2 College of Veterinary Medicine, Kyungpook National University, Daegu, 42566 Republic of Korea; 3 Institute of Agriculture and Life Science, Gyeongsang National University, Jinju, 52828 Republic of Korea

**Keywords:** *B. abortus*, Heat stress, Macrophage, NF-κB, ROS

## Abstract

**Background:**

*Brucella* causes a chronic and debilitating infection that leads to great economic losses and a public health burden. In this study, we demonstrated the brucellacidal effect of heat shock mediated by the induction of pro-inflammatory cytokines, reactive oxygen species (ROS) accumulation and apoptosis in murine macrophages and in mice.

**Results:**

RAW264.7 cells were incubated at 43 °C, and BALB/c mice were subjected to whole body hyperthermia. The data showed a reduction in bacterial survival in the mice after daily heat exposure. This was accompanied by increased levels of cytokines TNF, IL-6, IL-1β and IFN-γ in the sera of the mice. Gene expression of NF-κB and inducible nitric oxide production were also induced in the mouse splenic cells. In parallel with the bacterial reduction in the mouse model, an increased bactericidal effect was observed in RAW264.7 cells after exposure to heat stress. In addition, the heat stress increased both the nuclear translocation of NF-κB and the expression of the heat shock proteins HSP70 and HSP90 in murine macrophages. Furthermore, heat exposure induced the increase of pro-inflammatory cytokines, ROS accumulation and apoptosis but did not affect the production of nitric oxide (NO) in macrophages.

**Conclusion:**

This study demonstrated the induction of innate immune responses by heat stress that significantly reduced the intracellular survival of *B. abortus* in vitro and in vivo. Transcriptional factor NF-κB, which is a master regulator, could be termed a key activator of heat-induced immunity against *Brucella*. The increase in the expression and activation of NF-κB in splenic cells and macrophages was followed by enhanced antimicrobial effectors, including cytokines, ROS and NO that may contribute to the reduction of bacterial survival.

## Background

The ability to resist and modify cellular activities for survival during stress is a fundamental property of eukaryotes and prokaryotes. Particular stressors, including heat shock, can elicit a stress response that affects an organism’s immunity against a particular pathogen. Previous studies demonstrated a protective role of heat shock against sepsis and acute lung injury [1, 2]. Heat shock can modulate the signal transduction associated with the pro-inflammatory responses. The heat shock response has been associated with the induction of heat shock proteins, and it is one of the proposed mechanisms by which pro-inflammatory responses are modulated in favor of the host defense. However, the effect of heat stress in the context of *Brucella* infection is not yet elucidated. Some studies suggest that heat stress can activate pathogen recognition receptors in antigen-presenting cells, including macrophages, which are important for the recognition of pathogen-associated molecular patterns [3]. Heat shock induced upregulation of these receptors might lead to a heightened response to invading pathogens [3]. The activation of many Toll-like receptor complexes specific for bacterial lipopolysaccharides and expressed on macrophages triggers the induction of inflammatory genes as well as cytokines [4]. Toll-like receptor signaling pathways all lead to the activation of the nuclear factor-kappaB (NF-κB), which plays a critical role in orchestrating the expression of inflammatory genes [5, 6]. Interestingly, one study demonstrated the activation of NF-κB in rat liver after subjecting rats to heat stress [7], and another study showed the upregulation of the NF-κB pathway in monocytes under heat stress [3].

Macrophages play a major role in innate immunity. These cells recognize and respond to elevation in temperature and danger signals coming from inflammatory sites via pattern recognition receptors that eventually lead to the expression of heat shock proteins, antimicrobial products, as well as cytokine production [8].

From this premise, we try to evaluate the effects of heat stress through simulation via the heat-stress protocol that may mediate inflammation and subsequently influence brucellacidal activity.

In the present study, a stress response in murine macrophages and in mice subjected to *B. abortus* 544 infection was evaluated. In previous studies, macrophages exposed to heat stress displayed a greater capacity for phagocytosis and bacterial killing via rapid phagosome maturation against infection by group B *Streptococcus* [9]. Elevated temperature was the first stimulus discovered to induce heat shock proteins and other factors. Thermal stress triggers an intricate biochemical adaptive mechanism and gene expression that is favorable to the survival of the host. Hyperthermia can cause changes in gene expression. Typically, the exposure of mammalian cells in vitro to temperatures between 42 °C and 45 °C can induce heat shock; however, the results may be variable according to the type of tissue used [10]. Heat shock can be induced in the mouse by a heat shock protocol (whole body hyperthermia) followed by reversion to normal temperature at 37 °C [11].

*Brucella* infection remains one of the most significant zoonoses in the world. It causes a chronic and debilitating infection that leads to great economic losses and public health burdens [12]. The Centers for Disease Control and Prevention (CDC; USA) includes *Brucella* spp. in the select agent list because of its significant infection rate. Infection can be acquired via the respiratory and gastrointestinal routes from infected tissues as well as by ingestion of unpasteurized products [13].

*Brucella* spp. have the ability to circumvent the killing mechanisms of the host [14]. The chronicity of infection is due to the strategies evolved by these species. One significant feature is the ability to inhibit the induction of host pro-inflammatory responses. In one study, the lack of leukocyte recruitment as well as cellular and humoral responses was demonstrated in a mouse model. To cause this effect, the pathogen can invade the host cell prior to the development of defensive host mechanisms. Thus, a minimal degree of pro-inflammatory responses leads to the protection of intracellular *Brucella* [15]. Another mechanism used by *Brucella* is the inhibition of apoptosis of infected macrophages to maintain a protective environment that sustains a niche for its intracellular growth [15]. In this study, we evaluated the expression of cleaved caspase-3, which is considered to be a key effector caspase, being activated by caspase-8 and caspase-9. The activity of the caspases regulates the biochemical events that lead to the disassembly and eventual death of cells. The initiation of apoptosis is either via the mitochondrial or the death-receptor pathway mediated through caspase-9 and caspase-8, respectively, which in turn activate the main effector, caspase-3 [16].

The effect of heat stress on the expression of antimicrobial effectors was also evaluated. The regulation of mammalian immune responses has been reported to be influenced by the expression of nitric oxide (NO). As shown in previous studies, NO plays a role in *Brucella* infection [17]. Macrophage-mediated cytotoxicity is involved as an antimicrobial mechanism. Macrophages are also capable of eliciting regulatory molecules, including reactive oxygen species, which can regulate the intracellular growth of *B. abortus* [18].

## Results

### Exposing mice to heat shock restricts bacterial infection

**Fig. 1:**
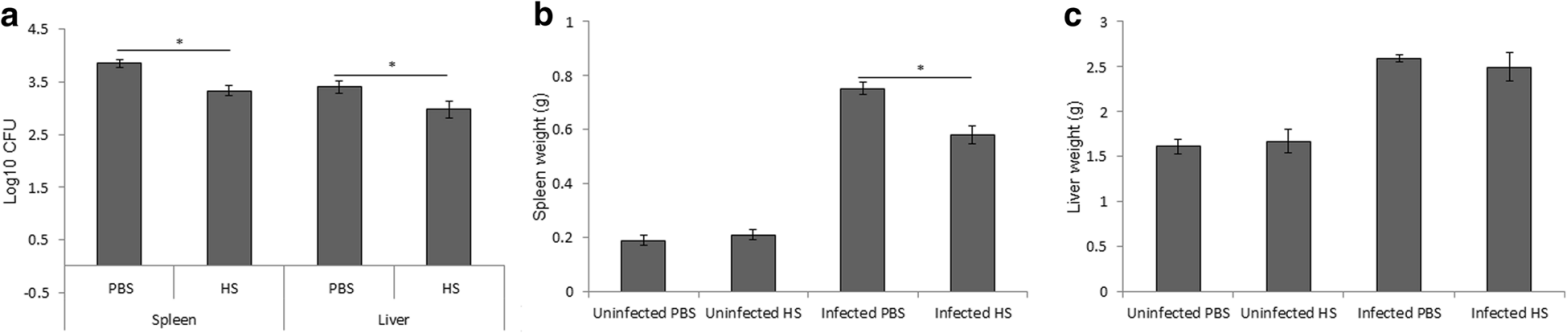
Daily heat exposure restricted bacterial survival in mice. The bacterial burdens in the spleen and liver (**a**), weight of spleen (**b**) and liver (**c**) were evaluated two weeks post-infection in heat-exposed and non-exposed mice. Data represent the mean ± SD of replicates of six samples. Asterisks indicate significant differences (*p <* 0.05)

Mice were sacrificed 14 days post-infection. The spleens and livers were collected and weighed. Daily exposure of mice to heat stress for 1.5 h after the initial infection on day 1 resulted in a reduced bacterial burden in the spleen and liver compared to control mice (Fig. 1a). The spleen weight of the treated mice was significantly different from that of the control mice; however, there was no significant difference in the liver weight (Fig. 1b, c).

### Heat stress induced cytokine production in splenic cells and sera of mice

**Fig. 2:**
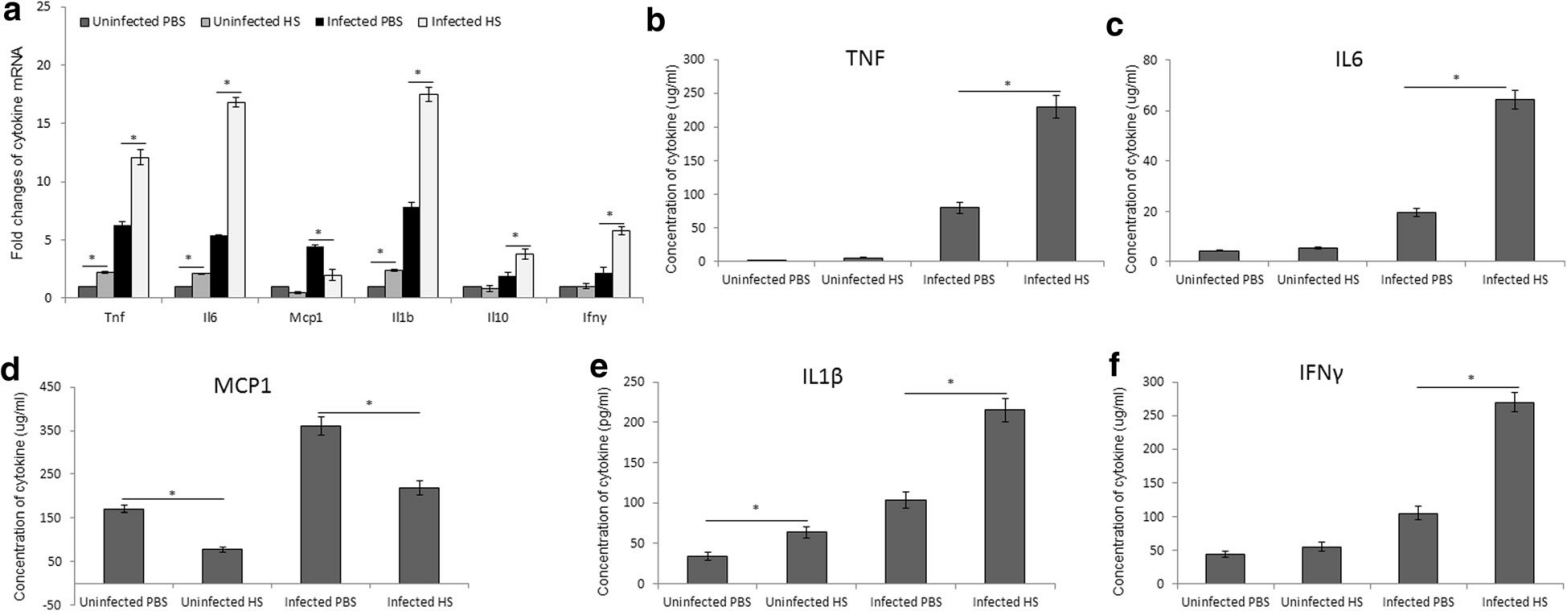
Heat stress induced cytokine secretion. Expression of cytokines *Il6, Il1b, Tnf, Mcp1, Ifng* and *Il10* by mouse splenic cells with and without exposure to heat quantified by RT-PCR (**a**). Quantification of cytokines: TNF (**b**), IL-6 (**c**), IL-1β (**d**), MCP-1 (**e**), and IFN-γ (**f**) in the sera of mice by flow cytometer and sandwich ELISA. Data represent the mean ± SD of replicates of six samples. Asterisks indicate significant differences (*p <* 0.05)

Cytokine expression in mouse splenic cells and their concentrations in sera were evaluated through RT-PCR and FACS or sandwich ELISA, respectively. An increased expression of *Tnf, Il6, Il1b, Ifng* and *Il10* was apparent in splenic cells of infected mice exposed to heat; however, *Mcp1* was decreased (Fig. 2a). In parallel, the production of TNF (Fig. 2b), IL-6 (Fig. 2c), IL-1β (Fig. 2d), and IFN-γ (Fig. 2f) was also elevated, and the level of monocyte chemoattractant protein-1 (MCP-1) was decreased in the infected group (Fig. 2e).

### Heat stress caused the increase of NF-κB and iNOS gene expression in splenic cells

**Fig. 3:**
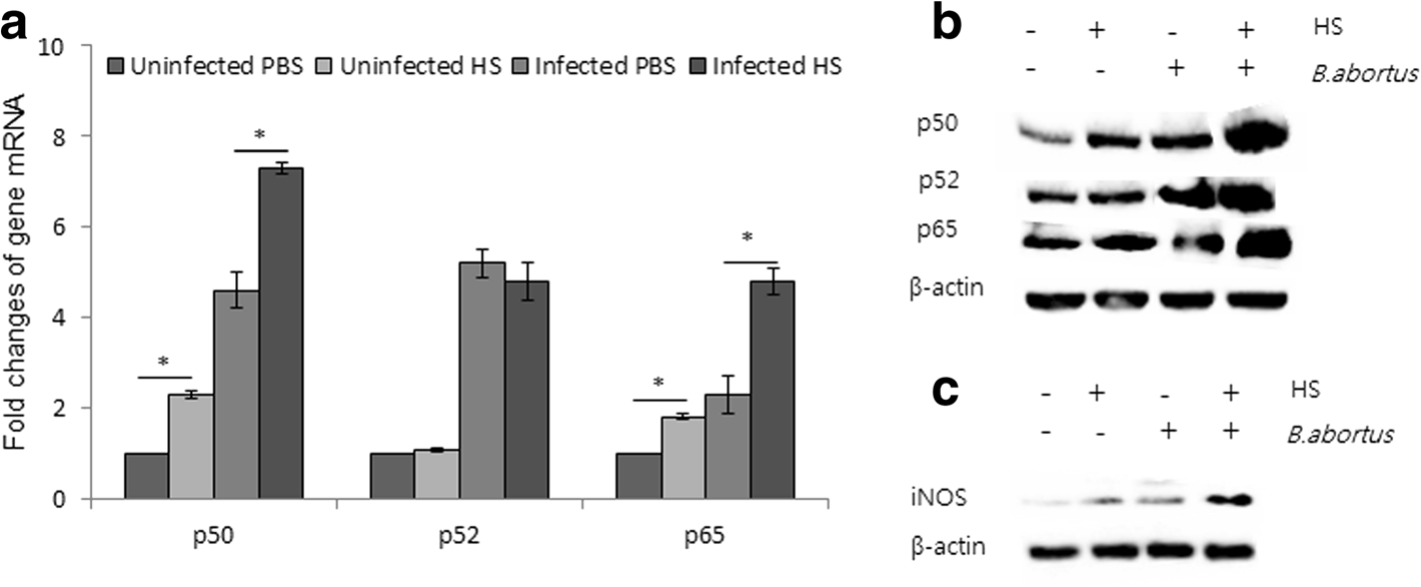
Heat stress caused an increase in NF- κB and iNOS expression in splenic cells. The expression of heterodimers of NF- κB: p50, p52 and p65 from splenic cells was quantified by RT-PCR (**a**). Immunoblot analysis of the expression of heterodimers of NFkB: p50, p52 and p65 from splenic cells (**b**). Immunoblot analysis of iNOS expression following heat exposure (**c**). Asterisks indicate significant differences (*p <* 0.05)

NF-κB is a central transcription factor responsible for controlling the expression of multiple cytokine genes involved in inflammatory responses, including TNF, IL-1, IL-6, IL-8 and IL-12, as well as NO production. To clarify the role of NF-κB in the heat shock response, the expression of the NF-κB subunit was investigated. Intriguingly, we found that members of the NF-κB family of transcription factors, p50 and p65, in infected splenic cells were upregulated under heat shock. In contrast, p52 was unchanged compared with the control (Fig. 3a). Western blot analysis showed that all three transcription factors were significantly increased in splenic cells infected and exposed to heat stress (Fig. 3b). This was accompanied by an upregulation of inducible nitric oxide synthase (iNOS) (Fig. 3c).

### Bacterial killing activity evaluated in murine macrophages exposed to heat stress

**Fig. 4:**
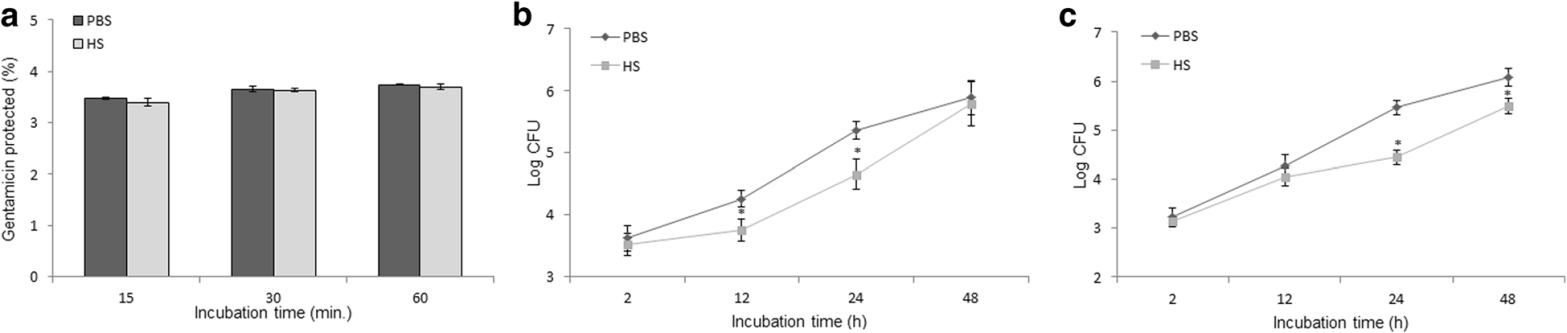
Bacterial killing activity was evaluated in murine macrophages exposed to heat stress. Bacterial internalization of *B. abortus* after treatment with heat stress (**a**). Intracellular growth of *B. abortus* in RAW 264.7 cells initially infected and exposed to heat (**b**). Intracellular growth of *B. abortus* in BMM initially infected and exposed to heat (**c**). Data represent the mean ± SD of triplicate experiments. Asterisks indicate significant differences (*p <* 0.01)

To evaluate the effect of heat stress-induced responses on bacterial pathogenesis, macrophages were pretreated prior to infection. As shown in Fig. 4a, bacterial internalization of *B. abortus* was not affected by pretreatment with heat. However, a significant reduction in the intracellular growth of *B. abortus* was observed after infection followed by heat exposure. This reduction was maintained up to 24 h post-infection (Fig. 4b). Consistent with these data, bone marrow-derived macrophages (BMM) subjected to hyperthermia were also shown to enhance the resistance to *B. abortus* infection up to 48 h post-infection compared to the control (Fig. 4c).

### Heat stress accelerated heat shock protein expression and NF-κB nuclear translocation but not NO production

**Fig. 5:**
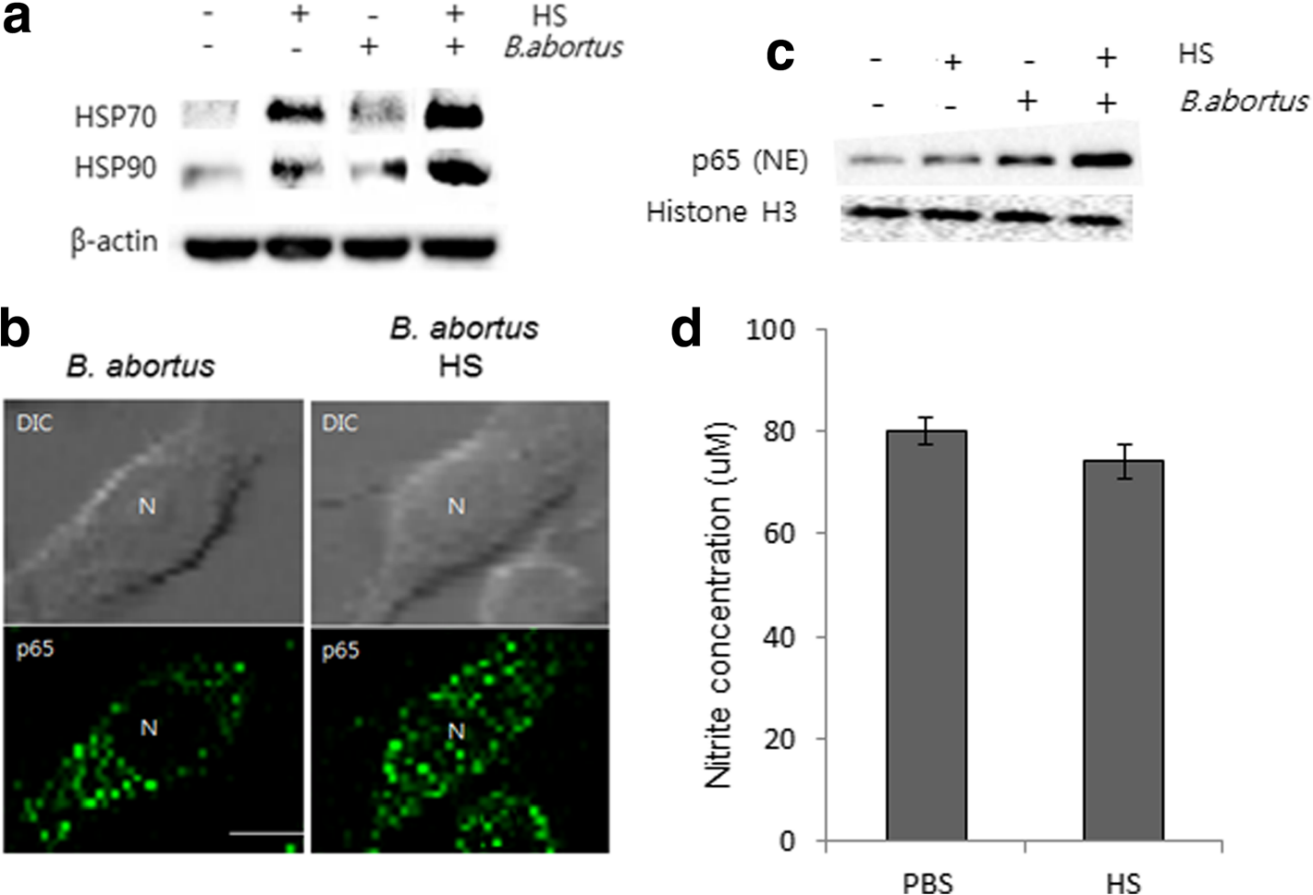
Heat stress accelerated heat shock protein expression and NF- κB nuclear translocation but not NO production at 24 h pi. The heat shock response induced production of heat shock proteins (HSPs) (**a**), NF- κB p65 translocation (**b**), and immunoblot analysis of cytoplasmic and nuclear NF- κB p65 following heat exposure (**c**). Effect of heat stress on nitrite production as quantified by the Griess assay (**d**). Data represent the mean ± SD of triplicate experiments. (Scale bar = 5 μm)

Heat shock proteins (HSPs) which are induced in response to heat stress were found to be a stimulator of antigen presentation process and immune response activation in immune cells. Particularly, HSP 90 and 70 were shown to interact with Toll-like receptor (TLR) 2 and 4 that induce the activation of NF-*k*B pathway leading to subsequent production of pro-inflammatory cytokines (TNF and IL-12) and antimicrobial effector NO [19] . Interestingly, in this study, we found that heat exposure induced a marked expression of HSPs (Fig. 5a) and NF-*k*B activation (Fig. 5b, c) in infected macrophages which was consistent with observation in splenic cells; however it did not influence the production of NO (Fig. 5d) in *B. abortus*-infected RAW 264.7 cells.

### Heat stress induces pro-inflammatory cytokine expression, ROS production and apoptosis in *B. abortus*-infected macrophages

**Fig. 6:**
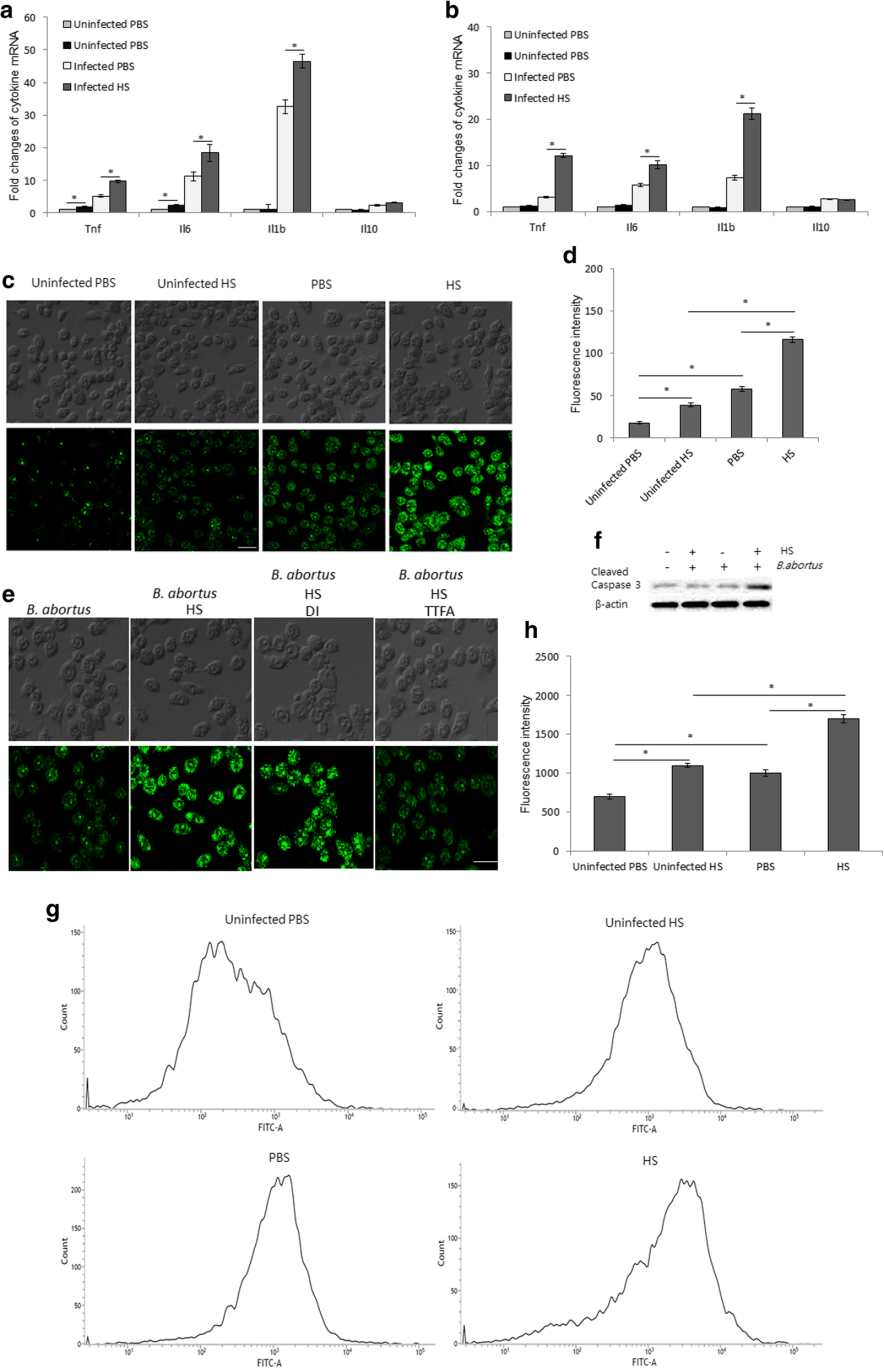
The effect of heat stress on the production of pro-inflammatory cytokines, ROS accumulation and apoptosis. The cells were infected with *B. abortus* and subjected to heat exposure. The production of pro-inflammatory cytokines and ROS was evaluated at 24 h pi while the apoptosis assay was performed at 48 h pi. Levels of pro-inflammatory cytokines: *Tnf*, *Il6*, *Il10*, *Il1b* and *Mcp1* in RAW 264.7 (**a**) and BMM (**b**) through RT-PCR. Production of ROS after heat exposure was measured by fluorescence microscopy (**c**) and spectrometry (**d**). The infected cells were treated with inhibitors of mitochondrial respiratory chain (TTFA) or NADPH oxidase (DI) (**e**). The apoptosis was evaluated by activation of caspase-3 (**f**) and flow cytometry (**g**) after heat exposure. The intensity of FITC was also evaluated from random 10,000 cells (**h**). Data represent the mean ± SD of triplicate experiments. Asterisks indicate significant differences (*p <* 0.01). (Scale bar = 20 μm)

Infected macrophages with or without heat stress were subjected to cytokine mRNA measurements, ROS quantification and evaluation of Casp-3 activation. Pro-inflammatory cytokines, including TNF, IL-6 and IL-1β but not IL-10, were significantly elevated under heat stress in both of RAW 267.4 (Fig. 6a) and BMM (Fig. 6b) cells. In addition, the RAW 267.4 cells also exhibited increased production of ROS (Fig. 6c, d) and upregulation of cleaved caspase-3 (Fig. 6f). To further identify the source of heat stress-induced intracellular ROS accumulation in infected RAW 264.7 cells, we treated cells with either thenoyltrifluoroacetone (TTFA) or diphenylene iodinium (DI) which are inhibitors of mitochondrial respiratory chain and NADPH oxidase (NOX), respectively. Interestingly, the treatment with TTFA, but not DI significantly inhibits the heat stress-induced intracellular ROS accumulation in infected cells (Fig. 6e). To complement the activation of caspase-3, we further evaluated apoptosis by flow cytometry assay. As expected, the heat exposure induced the apoptosis in *B. abortus*-infected RAW 264.7 cells (Fig. 6g, h).

## Discussion

This study demonstrated an enhancement of brucellacidal effect in vivo and in vitro mediated by the upregulation of the transcription factor, NF-κB and the subsequent induction of pro-inflammatory cytokines. Invading pathogens are attacked by the innate immunity as the first line of defense. Recognition of infectious agents by macrophages through toll-like receptors can initiate the signaling of the NF-κB cascade. One stealthy mechanism of *Brucella* is its ability to cause very minimal inflammatory responses compared with other bacterial infections at the onset of infection enabling it to persist chronically**.** Recognition of a pathogen-associated molecular pattern is the major requirement for the induction of inflammatory responses. One study demonstrated the upregulation of TLR2 and TLR4 in monocytes induced by heat stress [3]. These receptors have been observed to be highly expressed peaking within 9 h and 6 h, respectively, after heat exposure. Previous studies suggested that activation of these receptors might be an independent mechanism because it precedes the induction of heat shock proteins [3]. The induction of the heat shock response prior to any subsequent stress, including infections, has been demonstrated to be beneficial; however, the exact mechanism has not been elucidated [20]. Likewise, we suggest that the upregulation of NF-κB and the induction of proinflammatory cytokines manifested in this study are possibly influenced by the effect of heat stress on these toll like receptors as demonstrated in various studies showing upregulation of TLR2 and TLR4 [3]. The participation of TLRs; TLR2 and TLR4 in the recognition of *Brucella* infection has been documented in several studies. TLR2 was proposed to be able to induce IL-6, IL-12, TNF-α, and IL-10 upon stimulating peritoneal macrophages with *B. abortus* lipoproteins, such as Omp16 and Omp19 [21]. Interestingly, the involvement of TLR4 with non-canonical *Brucella* LPS activates the induction of NF-κB, stimulate the maturation of dendritic cells in response to its interaction with the protein; lumazine synthase [22] as well as upregulate the expression of co-stimulatory molecules and major histocompatibility class II (MHCII) in addition to the expression of TNF-α, IL-12p70 and IL-6 [23].

Previous studies have shown enhanced phagocytosis and the bacterial killing of group B *Streptococcus* in macrophages that were subjected to heat shock [9]; however, in the present study, pretreating macrophages with heat prior to *Brucella* infection did not cause any effect on the rate of bacterial internalization compared with the control. In contrast, intracellular growth was restricted up to 24 h after infection, suggesting that the heat shock response in murine macrophages might affect the signaling of receptors during the course of infection even when subjected to heat exposure post-infection with *Brucella*. In parallel to the in vitro result, the mouse model demonstrated reduced survival of *Brucella* after two weeks of daily exposure to heat.

Inflammation can activate NF-κB as demonstrated in several diseases. The expression of NF-κB plays an important role in the defense against invading pathogens. The NF-κB family is composed of five critical regulatory transcription factors in mammalian cells: RelA (p65), RelB, p105 (the precursor of p50), p100 (the precursor of p52) and c-Rel. These proteins form either homo or heterodimers among themselves that can regulate the expression of distinguishable but co-occurring genes responsible for stress responses, including genes encoding heat shock proteins and genes involved in inflammation, innate and adaptive immunity, anti-apoptosis, proliferation and cancer progression [24, 25]. The heterodimer RelA-p50 is the most active form associated with TLR signaling [5] and interestingly, was the most highly expressed among the p52 and p65 dimers in the infected splenic cells subjected to heat. Under normal conditions, in the absence of stimulation, sequestration of NF-κB in the cytoplasm is controlled by inhibitor proteins called Iκβ proteins. The inactive form of NF-κB can be made active by translocation to the nucleus [5]. This important event might be responsible for the induction of pro-inflammatory cytokines, including IL6 that can be induced by p50 and p65 in the rheumatoid arthritis pathology, as previously reported. In human monocytes, heterodimers of p65 and p50 activate the pro-inflammatory genes TNF-α and IL-1β [6] as well as other molecules and enzymes, including acute phase proteins and iNOS, which can have a favorable effect on cell survival in the presence of bacterial pathogens.

Interestingly, in this study, we found that the nuclear translocation and expression of nuclear p65 in murine macrophages is prominent after heat treatment and is followed by a marked increase in the production of important cytokines, including TNF, IL-6 and IL-1β but not IL-10. This is consistent with a study reporting that heat stressed macrophages demonstrated an increased response to lipopolysaccharide (LPS) treatment followed by NF-κB translocation [26].

In Gram-negative infections, accelerated recognition of LPS by mammalian cells, particularly macrophages, leads to a heightened response against LPS and lipoproteins and subsequent release of pro-inflammatory mediators, including IL-6, IL-12, IL-1β and TNF-α. This response benefits the host by enhancing inflammation at moderate levels, priming an enhanced immunity for the clearance of invading pathogens [27].

Our results showed significant elevation of the pro-inflammatory cytokines TNF, IFN-γ, IL-6 and, more importantly, IL-1β in the sera and splenic cells of mice exposed to heat stress. In addition, the effect of heat stress on the expression of another killing effector, NO, was determined. The results showed upregulation of the iNOS gene in splenic cells of heat-stressed *Brucella*-infected mice but not NO production in murine macrophages. NO can regulate the immune response in mammalian cells, and it acts on intracellular pathogens and an array of other infectious agents, including viruses and fungi. The role of NO as a significant molecule in *Brucella* infection is well established [16]. NO is produced by the enzyme NO synthase, and its antimicrobial effect depends on macrophage-mediated cytotoxicity. In particular, macrophages release regulatory molecules, including reactive oxygen intermediates, which have a role to play in the control of the growth of *B. abortus* [18]. The expression of the inducible form of iNOS is responsible for the massive induction of NO, and it is induced by pro-inflammatory cytokines, including TNF-α, IL-1 and IFN-γ [28]. It has also been shown that endogenous TNF-α is required for the production of reactive oxygen intermediates that confer anti-*Brucella* activities to macrophages [29]. The significant elevation of ROS in macrophages might contribute to the clearance of *Brucella* observed in this study.

Among several cytokines, IFN-γ and TNF-α are key to *Brucella* infection. The IFN-γ-mediated Th1 immune response is thought to be essential to the control of *Brucella* infection, and in its absence, there is diminished bacterial clearance [30]. In contrast, production of IL-10, a counter-regulator of IFN-γ during the early onset of infection, is beneficial for the pathogen, promoting intracellular replication by setting a level conducive for the survival of the pathogen [18]. In this study, however, heat stress did not induce significant differences in IL-10 in splenic cells and sera from infected mice and from the controls. IL-1β induces the release of other pro-inflammatory cytokines, including TNF and IL-6, and activates the Th17 bias of the cellular adaptive responses [31]. In turn, IL-6 is highly elevated, consistent with previous articles, which reported that IL-6 could be detected in the sera of mice after endotoxin challenge [26]. In contrast, MCP-1 is attenuated by heat stress in both sera and splenic cells, but it is required for monocyte recruitment from bone marrow to the site of inflammation [32].

As previously reported, increases in the cytokines TNF-α and IL-6 and the concurrent production of NO and iNOS have been correlated with the induction of HSP70 in heat-treated macrophages subjected to IFN-γ and LPS stimulation [33]. However, more investigations are required to elucidate the effect of heat stress on *Brucella* pathogenesis.

Another important virulence factor of *B. abortus* that contributes to the chronicity of this infection is its ability to inhibit macrophage apoptosis in infected cells. Sustaining macrophage viability provides an intracellular niche that is important in circumventing the antagonistic external environment and allowing an environment conducive to bacterial replication [34]*. Brucella infection will cause the expression of A1 of the blc2 family, which is an anti-apoptotic gene* [35]. Other bacteria, including *Yersinia* spp., utilizing the deubiquitinating protease YopJ were documented to be able to retain IκB sequestered in the cytoplasm, inhibiting nuclear translocation and the subsequent prevention of the induction of protective pro-inflammatory cytokines [36]. The upregulation of the subunits of NF-κB and the subsequent nuclear translocation under heat stress might contribute to counter the anti-apoptotic ability of *B. abortus* in addition to the enhancement of pro-inflammatory cytokines that are brucellacidal in nature.

In conclusion, the brucellacidal effect of heat stress demonstrated in this study might be attributed to several factors. One possible mechanism is the influence of heat stress in the upregulation of Toll-like receptors during infection, which may sustain and heighten responsiveness to *B. abortus* that leads to an enhanced response along the signal transduction cascade culminating in the activation of NF-κB, as demonstrated in this study. These events led to the induction of pro-inflammatory cytokines that restrict bacterial survival in macrophages and in the mice. NF-κB, in turn, is highly associated with the regulation of the transcription of heat shock proteins, which in itself can modulate the expression of transcription factors [10]. However, this is another aspect that requires further investigation.

This study illustrated the antimicrobial effects of heat stress in macrophages and mouse model in response to *B. abortus* infection through the activation of NF-κB pathway leading to the induction of pro-inflammatory cytokines, antimicrobial effectors (ROS and NO) and apoptosis. These effects results in an enhanced reduction in the intracellular survival of *B. abortus* in vitro and in vivo.

## Methods

### Bacteria and cell culture

*B. abortus* 544 (ATCC 23448), a smooth, virulent *B. abortus* biovar 1 strain was used in this study. It was cultivated in Brucella broth (Becton Dickinson, MD, USA) or on Brucella agar and grown at 37 °C with vigorous shaking until it reached the stationary phase. RAW 264.7, a murine cell line, was grown at 37 °C in 5% CO_2_ atmosphere in RPMI medium (Thermo Fisher Scientific, MA, USA) containing 10% heat-inactivated fetal bovine serum, with or without 100 U/ml penicillin and 100 μg/ml streptomycin (all purchased from Gibco, Invitrogen, CA, USA).

### BMM preparation

Bone marrow-derived macrophages (BMM) from female BALB/c mice were prepared and incubated at 37 °C with 5% CO_2_ atmosphere as previously described [37]. BMMs were cultured in cultivation dishes (100 mm × 20 mm) with 10 ml L-cell conditioned medium. After 5 days of incubation, 10 ml fresh medium without antibiotics was added and the cells were incubated further for 5 days. After 10 days of incubation, BMMs were washed three times with PBS and incubated with fresh RPMI 1640 medium containing 10% (*v*/v) heat-inactivated fetal bovine serum (FBS).

### Bacterial uptake and intracellular growth

To monitor bacterial uptake, internalization assay was performed as previously described [38]. Briefly, macrophages at 10^6^ cells per well were maintained in RPMI containing 10% (v/v) fetal bovine serum (RPMI/10% FBS) and subjected to 43 °C for 1.5 h following infection with bacteria at a multiplicity of infection (MOI) of 100, centrifuged at 150 x *g* for 10 min at room temperature and incubated at 37 °C in 5% CO_2._ At 0, 30 and 60 min post-infection, the infected cells were washed once with phosphate buffer saline (PBS) and then incubated in RPMI/10% FBS and gentamicin (30 μg/ml) for 30 min to kill extracellular bacteria. Finally, the cells were washed with PBS, lysed with distilled water and plated on Brucella agar.

To monitor intracellular growth, the macrophage maintained in RPMI/10% FBS were infected with *B. abortus* at MOI of 100 and incubated for 2 h at 37 °C. The cells were then transferred to 43 °C for 1.5 h and returned to 37 °C for further incubation. At 2, 24 or 48 h post-infection, RPMI/10% FBS containing gentamicin (30 μg/ml) was added and incubated for 30 min. The cells were then washed with PBS, lysed with distilled water and plated on Brucella agar.

### Infection of mice with *B. abortus* 544

Female BALB/c mice, 8 weeks old (Japan SLC, Japan) were randomly allocated into four groups with six mice per group (uninfected with and without heat shock, infected with and without heat shock) and housed in standard conditions with ad libitum access to food pellets and water. Acclimatization of one week preceded the intraperitoneal infection with *B. abortus* 544 at a concentration of 5 × 10^5^ colony forming unit (CFU)/mouse used. The exposure to heat shock is as previously reported [11] with few modifications. At 24 h post-infection, the mice were subjected daily to hyperthermia at 43 °C for 1.5 h and returned back to room temperature and daily for 13 days. They were sacrificed on the 14th day post-infection, and sera, spleens and livers were collected. The spleens and livers were weighed and spliced approximately 0.5 mg each sample for homogenizing in PBS. The homogenates were diluted 100-fold with PBS and plated on Brucella agar and then incubated for 3 days at 37 °C. The Log_10_ number of CFUs for each spleen sample was calculated.

### Total RNA extraction

DNase-treated RNA was isolated from spleens as previously described, following the manufacturer’s protocol [39]. Briefly, macrophages and splenic cells were subjected to RNA extraction using RiboEx reagent (Geneall, South Korea) and RNeasy® Mini kits (Qiagen, Germany). RNase-free DNase 1 (Qiagen, Korea) was used to remove genomic DNA contamination. The purified RNA was collected and stored at − 70 °C until use. Total RNA was measured using a nano spectrophotometer (Optizen, Korea).

### Quantitative real-time RT-PCR

Single-stranded cDNA was synthesized from 1 to 2 μg RNA using a Quantitect® Reverse Transcription Kit (Qiagen, Hilden, Germany) according to the manufacturer’s instructions. Real-time RT-PCR was performed in duplicate using a CFX96 real-time RT-PCR system (Bio-Rad, USA) with SYBR Green (Bioneer, Korea). Expression levels of target genes were determined using the primers shown in Table 1. Gene expression levels were quantified using ∆∆C_t_ [40]. The β-actin gene was used as the reference to normalize the relative expression levels of individual transcripts. The data were analyzed using the Bio-Rad CFX software.

**Table 1:** Primer sequences of macrophage genes used for qRT-PCR assays

Gene name	Forward primer	Reverse primer
*b-actin*	5’-CGCCACCAGTTCGCCATGGA-3′	5’-TACAGCCCGGGGAGCATCGT-3
*Il1b*	5’-CAACCACACAAGTGATATTCTC-3′	5’-GGATCCACACTCTCCAGCTGC-3
*Il6*	5’-TCCAGTTGCCTTCTTGGGAC-3’	5’-GTACTCCAGAAGACCAGAG-3’
*Tnf*	5’-CACAGAAAGCATGATCCGCGA-3’	5’-CGGCAGAGAGGAGGTTGACTT-3’
*Il10*	5’-TGGCCCAGAAATCAAGGAGC-3’	5’-CAGCAGACTCAATACACACT-3’
*Ifng*	5’-TGAACGCTACACACTGCATCT-3’	5’-CGACTCCTTTTCCGCTTCCTG-3’
*Mcp1*	5′- GGTCCCTGTCATGCTTCTGGG-3’	5’-TCCAGCCTACTCATTGGGATC-3’
*P50*	5’-CTCACTCAATATTTAATGCAG-3’	5’-CCCTCCGTGTGATGGGCCTTC-3’
*P52*	5’-CCCATGGAGGTTTGCCAGGTG-3’	5’-CCCACCAGACTGTGGGCATGC-3’
*P65*	5’-CATCCACATGAACTTGTGGGG-3’	5’-CTGGCTAATGGCTTGCTCCAG − 3’

### Total protein extraction from spleen

The total protein from spleen was extracted by RIPA buffer (sc-24,948, Santa Cruz Biotechnology, Texas, USA) in following the manufacturer’s protocol. Briefly, frozen spleens were thawed and homogenized in RIPA buffer containing protease inhibitor cocktail (PIC). The lysates were then centrifuged at 10,000 x *g* at 4 °C for 10 min and the supernatant containing total proteins was collected.

### Collection of nuclear protein extracts from macrophages

Macrophages were prepared with cold PBS wash and collected with a cell scraper and centrifuged at 1500 x *g* at 8 °C for 5 min. Buffer A (10 mM HEPES, pH 7.9, 10 mM KCl, 1.5 mM MgCl_2_, 1 mM DTT) was used to resuspend the cells, and the suspension was frozen in a dry-acetone bath. Cells were thawed in an ice bath followed by centrifugation at 1200 x *g* at 4 °C for 10 min. The supernatant containing the cytoplasmic extracts and the pellet containing the nuclei were separated. Buffer C (20 mM HEPES, pH 7.9, 0.4 M NaCl, 1.5 mM MgCl_2_, 25% glycerol, 0.2 mM EDTA, 1 mM DTT), supplemented with 0.5 mM phenylmethylsulfonyl fluoride (PMSF, Sigma-Aldrich, CA, USA) protease inhibitor was used to resuspend the pellet. This suspension was incubated at 4 °C for 30 min with gentle stirring in an angular position and then centrifuged at 20,000 x *g* at 4 °C for 20 min. The supernatant collected from this suspension was the final nuclear extract, which was added to Buffer D (20 mM HEPES, pH 7.9, 50 mM KCl, 25% glycerol, 0.2 mM EDTA, 1 mM DTT) supplemented with 0.5 mM PMSF. The same buffer was added to the cytoplasmic extract, and both extracts were stored at − 70 °C. Protein concentrations were determined with the bicinchoninic acid reaction (BCA, Pierce, Rockford, IL) [41].

### Immunoblot analysis

Immunoblot analysis was carried out according to the previously reported method [42]. Proteins extracted from RAW 264.7 cells and splenic cells as well as from nuclear extracts from macrophages were evaluated for a variety of proteins. Cells were cultured in 6-well plates, infected with *B. abortus* for the indicated time, incubated at 37 °C for 2 h, followed by incubation at 43 °C for 1.5 h and returned back to 37 °C for 24 h. Cells were washed with ice-cold PBS twice and lysed with ice-cold radioimmunoprecipitation assay (RIPA) buffer containing 1% protease inhibitor cocktail for 30 min at 4 °C. Samples were separated by SDS-PAGE. The proteins were transferred to Immobilon-P membranes (Millipore, USA) using 1X transfer buffer (25 mM Tris, 192 mM glycine and 20% methanol) with a constant current of 2 mA/cm^2^ for 1.5 h in a semi-dry electroblot assembly (Bio-Rad, USA). The membrane was blocked with 5% (*w*/*v*) skim milk in 1X Tris-buffered saline-Tween 20 (TBS-T) (20 mM Tris-HCl, 150 mM NaCl, Tween 0.1%, pH 7.6) for 30 min at room temperature. The membranes were incubated with phospho-specific antibodies against HSP70, HSP90, intact or cleaved Caspase 3, p50, p52, p65 or iNOS overnight at 4 °C. All antibodies were obtained from Cell Signaling (USA). Binding of the primary antibody was detected with a horseradish peroxidase (HRP)-conjugated secondary antibody (Thermo Scientific, USA; 1:1000 dilution) in 5% blocking buffer for 1 h followed by a wash with 1X TBS-T. The signal was detected using a luminal-coumaric acid-H_2_O_2_ detection solution (Atto Corporation, Japan) and a Molecular Imager® ChemiDoc™ XRS+ system machine (Bio-Rad Laboratories, USA).

### Indirect immunofluorescence

RAW 264.7 macrophages at 10^6^ cells per well were infected with *B. abortus* and incubated for 24 h at 37 °C in 5% CO_2_. Cells were then fixed with 4% paraformaldehyde, incubated at 37 °C for 1 h, permeabilized with 0.1% Triton X-100 for 10 s at 4 °C and incubated with blocking buffer (2% goat serum in PBS) for 1 h. Rabbit anti-mouse NF- κB p65 and FITC-conjugated anti-rabbit IgG were subsequently added as primary and secondary antibodies (Santa Cruz), respectively. The cells were mounted with Permafluor mounting medium and analyzed by using a laser scanning confocal microscope (Olympus FV1000, Japan). The images were processed using FV10-ASW Viewer 3.1 software.

### ROS and NO detection

RAW 264.7 cells at 10^6^ cells per well were infected with *B. abortus* followed by heat exposure at 43 °C for 1.5 h and incubated for 24 h at 37 °C. The ROS content was then evaluated by fluorescence microscopy (Total ROS detection kit, ENZ-51011, Enzo Life Sciences) and spectrometry (DCFDA cellular ROS detection assay kit, Ab113851, Abcam) according to the manufacturer’s instructions. The nitrite concentration of culture supernatants was also assessed by the Griess reaction (Sigma).

### Cytokine quantitation

The levels of IL-6, IL-10, IFN-γ, TNF and MCP-1 in serum samples were determined by cytometric bead arrays (BD CBA Mouse Inflammation Kit, USA), and the level of IL-1β in serum samples was determined by sandwich ELISAs (Abcam, USA) following the manufacturer’s protocol.

### Flow cytometry for apoptosis

The apoptosis was evaluated by flow cytometry using Apoptosis detection kit (Abcam) at 48 h pi in accordance with the manufacturer’s instructions. Briefly, infected macrophages at 10^6^ cells per well were exposed to 43 °C for 1.5 h, incubated for 48 h at 37 °C and then stained with apopxin green. The apoptosis was determined from 10,000 events.

### Statistical analysis

Data were statistically analyzed using the Student’s t-test or ANOVA with Tukey’s HSD (Honestly Significant Difference) exact test to compare differences between the groups. Data were considered statistically significant if *p* < 0.05. Data were expressed as the mean ± standard error (SE).

## **Abbreviations**

BCA: bicinchoninic acid
CDC: Centers for Disease Control and Prevention
CFU: colony forming unit
DMEM: Dulbecco’s modified eagle medium
FBS: fetal bovine serum
HSP: heat shock protein
IFN: interferon
IL: interleukin
iNOS: inducible nitric oxide synthase
LPS: lipopolysaccharide
MCP-1: monocyte chemoattractant protein-1
MOI: multiplicity of infection
NF: nuclear factor
NO: nitric oxide
PBS: phosphate buffer saline
PMSF: phenylmethylsulfonyl fluoride
RIPA: radioimmunoprecipitation assay
ROS: reactive oxygen species
TLR: Toll-like receptor
TNF: tumor necrosis factor
